# CPITN changes during pregnancy and maternal demographic factors ‘impact on periodontal health

**Published:** 2015-02

**Authors:** Fahimeh Rashidi Maybodi, Ahmad Haerian-Ardakani, Farzaneh Vaziri, Arezoo Khabbazian, Salem Mohammadi-Asl

**Affiliations:** 1 *Department of Periodontology, Faculty of Dentistry, Shahid Sadoughi University of Medical Sciences, Yazd, Iran.*; 2 *Dr. Mohammadi-Asl Clinic, Soosangerd, Iran. *

**Keywords:** *CPITN*, *Pregnancy*, *Periodontal status*, *Demographic*

## Abstract

**Background:**

There have been speculations about the effects of hormonal changes and socio-demographic factors on periodontal health during pregnancy.

**Objective:**

According to the lack of sufficient epidemiologic information about the periodontal status of pregnant women in Yazd, this study was accomplished to determine the changes of Community Periodontal Index for Treatment Needs (CPITN) during pregnancy and evaluating the possible relationship between this index and demographic characteristics of the mothers.

**Materials and Methods:**

This was a longitudinal descriptive study. The samples included 115 pregnant women who were referred to health centers of Yazd, Iran. The mothers’ data were obtained from a questionnaire consisted of 3 parts: consent paper, demographic data and CPITN records. Examination was performed with dental unit light, flat dental mirror and WHO’s scaled probe.

**Results:**

In the beginning of the study, 60.1% of checked sextants had healthy gingival status. 25.9% had code1 and 14% had code 2. Code 3 and 4 were not seen in any sextants. There was a significant relationship between lower CPITN and higher maternal education, occupation and more frequencies of tooth-brushing but there was not a relationship between CPITN and mother’s age and number of pregnancies. CPITN had a significant relationship with increasing of the gestational age.

**Conclusion:**

There might be a relationship between increasing the month of pregnancy and more periodontal treatment needs. CPITN Increasing during pregnancy shows the importance of periodontal cares during this period.

## Introduction

Periodontal disease is a multi-factorial infectious disease in which the normal balance between the microbial plaque and the host response is disturbed. Environmental, physical, chemical and social factors and stress are likely to affect this disease or alter its manifestations. Some systemic conditions may also contribute to the initiation and progression of gingivitis and periodontitis (1, 2). The bilateral relationship between periodontal disease and pregnancy has been known for many years. It is confirmed that an increased incidence of gingivitis may happen during pregnancy (3). 

The maternal periodontal disease can also be a potential independent risk factor for low birth weight babies and preterm delivery (4-8). In addition to effects of hormonal changes on periodontium, there are some speculations about the impact of demographic factors on periodontal health. For example, in some studies, the relationship between higher maternal age and increased Community Periodontal Index for Treatment Needs (CPITN) has been mentioned (9, 10). Yalcin *et al, Golpasand et al* and Wandera *et al* found that periodontal status in pregnant women may be influenced by cultural and social conditions such as their education level or their awareness about periodontal care (9-11). Some other studies pointed to the relationship between higher gestational age and increased CPITN (9, 10). The results of the study of Tezel *et al* also showed that CPITN increases with higher trimester of pregnancy (12). It is obvious that periodontal changes usually occur in this group of women and some factors make them more vulnerable to local irritations such as calculus and plaque formation which may lead to periodontal diseases (13). 

Thus the aim of the present study was to determine CPITN possible relationship with socio-demographic characteristics such as maternal age, education level and employment status. CPITN changes during pregnancy period were investigated too. CPITN could also indicate the probable periodontal treatments needs in mothers.

## Materials and methods

This is a analytic descriptive study. Ethical committee of Shahid Sadoughi Medical University has approved this study. One hundred and fifteen 18-35 year-old pregnant women who attended the health centers (Rahmat Abad, Kheyr Abad, Emam-shahr & Akbar Abad) in the city of Yazd between March 2013 and October 2013, were selected by random cluster sampling by using a table of numbers. 

By considering the inclusion criteria, the chosen women were non-smokers and they were in the first trimester of pregnancy and had no systemic problems (such as hormonal disorders, blood disorders, diabetes, cardiovascular diseases, rheumatoid arthritis, and connective tissue diseases) and did not take drugs affecting periodontium (such as corticosteroids and anti-hypertension drugs) and had no tendency to perform periodontal treatments before their delivery. All these criteria were checked by asking from mothers during their first examination.

The developed questionnaire for data collection was consisted of three parts: informed consent paper, demographic data (Age, parity, current month of pregnancy, educational level, employment status, and frequency of tooth brushing), and determination of CPITN score for periodontal treatment need assessment. These three parts were completed by the examiner. A code number was allocated for each patient ad no name was recorded. Examination was performed with dental unit light, flat dental mirror and WHO scaled probe. For assessing the need for periodontal treatment (using CPITN index) in pregnant women, the dentition was divided into six sextants (one anterior and two posterior regions in each dental arch). The periodontal conditions are scored as follows:

- Code 0 is given to a sextant with no sign of pocket or calculus and bleeding on probing (gingival health with no treatment needs).
- Code 1 is given to a sextant with no pockets, calculus or overhangs of fillings but in which bleeding occurs after gentle probing in one or several gingival units (mild gingivitis; improvement of oral hygiene is needed).
- Code 2 is assigned to a sextant if there are no pockets exceeding 3 mm, but in which, dental calculus and plaque-retaining factors are seen or recognized in sub-gingival regions (established gingivitis; scaling, removal of overhangs, and improvement of oral hygiene is needed).
- Code 3 is given to a sextant that harbors 4-5 mm deep pockets (mild periodontitis, scaling, removal of overhangs, and improvement of oral hygiene is needed).
- Code 4 is given to a sextant that harbors pockets 6 mm deep or deeper (periodontitis and complex treatments such as surgery is needed).

Examination started from the right maxillary sextant and then reached to the middle and then left maxillary one. Mandible examination began from left and ended at right posterior sextant. A sextant was examined separately if only there were at least 2 maintainable teeth in it. Otherwise it was considered as a part of its adjacent sextant. To determine the pocket depth, 6 points around each tooth were examined and the highest score in each sextant was recorded as that specific sextants’ code.

At baseline, the relationship between CPITN and maternal demographic characteristics such as age, education level, employment status, parity and frequency of tooth-brushing was investigated. Then examinations for each mother were repeated in fifth month (2^nd^ trimester) and eight months (3^rd^ trimester) of pregnancy to compare the changes of this index during this period. Women who did not come at the scheduled times (2^nd^ and 3^rd^ trimester of pregnancy) were supposed to be excluded from the study but all 115 mothers came for their gynecologist’s follow up observations and we could also examined them for ourselves purposes. 

After measuring the CPITN in the third trimester, oral health instructions and required information about the use of dental floss and mouthwashes were given to all participants and if there was a need for periodontal treatment interventions in any of the mothers, they were referred to periodontology department of Shahid Sadoughi dental faculty.

**Statistical analysis**

Collected data were analyzed by SPSS 16 software (Statistical Package for Social Sciences, version 16, SPSS Inc. USA) and Chi-square test and Student’s *t*-test. A probability value of <0.05 was taken as statistically significant.

## Results

In this study, 115 pregnant women with an average age of 26.28±4.03 participated. Demographic information of pregnant women in the study is shown in table I. Among all examined sextants of participants, there were a total of 11 edentulous Sextants which were not included in the study and findings were reported on the base of 679 remained Sextants. At baseline, 408 Sextants (60.1%) were healthy and had code 0, 176 sextants (25.9%) had code 1 and 95 Sextants (14%) had code 2 and no Sextants with codes 3 and 4 were observed.

As can be seen in table II, the initial CPITNs in different age groups were compared with each other by Chi-square test and no significant relationship between this index and age was indicated (p=0.36) (Table II). Code zero, which indicates healthy periodontal status was more frequent in pregnant women with education level higher than diploma (72.2%) and Codes 1 and 2 had higher frequency in illiterate women. Code 3 and 4 were not observed in any of the groups. Chi-square test showed statistical difference between CPITN’s decrease in pregnant women and higher education (p=0.001) (Table II). 

Pregnant women were divided into two groups of employed and housekeeper. Although both groups had codes 0 and 1 as the most common codes in their own but employed group had more code 0 and code 1 and housekeepers’ group had more code 2 in comparison with other group. This difference was statistically significant (p=0.001) (Table II). As it is evident in table III, the frequency of the code 0, which is an indicator of periodontal health, was higher in women who brushed 3 times a day (78.2%) than those who had 1 or 2 times brushing a day (25.8%). The relationship between CPITN and higher frequency of brushing was statistically significant (p=0.001) (Table II). 

Pregnant women, who were in their first or second pregnancy, had lower frequency of code 2 than the women who were in their third or fourth pregnancy but code 2 were decreased in fifth pregnancy compared to fourth pregnancy. Thus, no significant association between CPITN and the parity was seen (p=0.12) (Table II). In this study, the CPITN indices in the first, second and third trimesters were compared and the results showed that CPITN increased as the month of pregnancy increased (Table III).

**Table I:** Distribution of participants on the basis of age, education level, and occupation

**Demographic data **			**Number**	**Percentage**
**Age**				
	Under 25 years-old		41	35.7
	25-30 years-old		52	45.2
	Over 30 years-old		22	19.1
**Education**				
	Illiterate		11	9.6
	Under Diploma		20	17.4
	Diploma		54	47
	Higher than Diploma		30	26
**Occupation**				
	Employed		92	80
	Housekeeper		23	20

**Table II:** Comparison of different codes of the first trimester CPITN’s frequency on the basis of demographic data, brushing frequency, and parity

		**First trimester CPITN **					**Total**	**p-value**
		**0**	**1**	**2**	**3**	**4**		
**Age**								
	Under 25 years-old	187 (60.5)	88 (28.5)	34 (11)	0 (0)	0 (0)	309 (100)	0.36
	25-30 years-old	159 (65.4)	55 (22.6)	29 (11.9)	0 (0)	0 (0)	243 (100)	
	Over 30 years-old	62 (48.8)	33 (26)	32 (25.2)	0 (0)	0 (0)	127 (100)	
**Education**								
	Illiterate	21 (35)	20 (33.3)	19 (31.7)	0 (0)	0 (0)	60 (100)	0.001
	Under Diploma	50 (43.1)	35 (30.2)	31 (26.7)	0 (0)	0 (0)	116 (100)	
	Diploma	207 (64.1)	81 (25.1)	35 (10.8)	0 (0)	0 (0)	323 (100)	
	Higher than Diploma	130 (72.2)	40 (22.2)	10 (5.6)	0 (0)	0 (0)	180 (100)	
**Occupation**								
	Housekeeper	3.8 (56.9)	143 (26.4)	90 (16.6)	0 (0)	0 (0)	541 (100)	0.001
	Employed	100 (72.5)	33 (23.9)	5 (3.6)	0 (0)	0 (0)	138 (100)	
**Brushing frequency**								
	1	117 (50.2)	56 (24)	60 (25.8)	0 (0)	0 (0)	233 (100)	0.001
	2	169 (58.3)	88 (30.3)	33 (11.4)	0 (0)	0 (0)	290 (100)	
	3	122 (78.2)	32 (20.5)	2 (1.3)	0 (0)	0 (0)	156 (100)	
**Parity**								
	1	185 (61.1)	80 (26.4)	38 (12.5)	0 (0)	0 (0)	303 (100)	0.12
	2	168 (62.5)	70 (26)	31 (11.5)	0 (0)	0 (0)	269 (100)	
	3	49 (53.8)	20 (22)	22 (24.2)	0 (0)	0 (0)	91 (100)	
	4	2 (18.2)	6 (54.5)	3 (27.3)	0 (0)	0 (0)	11 (100)	
	5	4 (80)	0 (0)	1 (20)	0 (0)	0 (0)	5 (100)	

Data are presented as n (%).

Chi-Square test

CPITN: Community Periodontal Index for Treatment Needs

* In housekeepers’ group, 11 edentulous sextants existed which were not considered as separated ones.

**Table III:** CPITN in different trimesters

**Trimester**	**CPITN**					**p-value**
	**0**	**1**	**2**	**3**	**4**	
First	408 (59.1)	176 (25.5)	95 (13.8)	0 (0)	0 (0)	0.018
Second	388 (57.1)	156 (23)	135 (19.8)	0 (0)	0 (0)	
Third	316 (46.5)	132 (19.4)	220 (32.4)	11 (1.6)	0 (0)	

Data are presented as n (%).

Chi-Square test

CPITN: Community Periodontal Index for Treatment Needs

## Discussion

In the present study, among 679 examined sextants, 60.1% of these sextants were healthy and 25.9% and 14% had code 1 and 2 respectively. No sextant with code 3 and 4 was observed. This can be a sign of fairly good hygiene performance in pregnant women, compared to the past. The results of two previous studies conducted in the city of Yazd showed that the oral health of pregnant women in Yazd has also been steadily improving in the interval between those studies (14, 15). 

In this study, the CPITN indices among pregnant women in three age groups (less than 25 years, 30-25 years and ≥30 years) were not significantly different with each other, but the fact that the sextants with zero code in women over age 30 were less than two younger age groups and the highest frequency of code 2 were seen in this age group, showed that the higher the age, the periodontal status gets worse. These results were in agreement with Karunachandra, Golpasand, Safavi, Nouri, and Wandera‘s studies (9, 10, 16-18). Vogt *et al* study in Brazil was also confirmed that the prevalence of periodontal disease is correlated with increasing gestational age and maternal age. Wandera *et al* study in Uganda showed that it is more probable that older pregnant women have higher CPITN scores (9, 19).

The results of this study showed that the zero code that indicates better gum status was more frequent in mothers with education level higher than diploma. Codes 1 and 2 had higher frequency in illiterate women. Thus, the relationship between CPITN and education level was significant (p=0.001) (Table II). This finding is consistent with the results of Paknejad, Torabi and Golpasand studies (10, 13, 20). It is expected that when the level of education is higher, awareness increases and periodontal status becomes better and CPITN scores decreases but Baghaei *et al* and Hosseini *et al* studies in Yazd did not show a significant relationship between oral health knowledge of pregnant women and their education level (14, 15). In this study, periodontal conditions in pregnant women who were employed were significantly better than housewives (p=0.001) which is consistent with the results of Golpasand, Safavi, Torabi and Yalcin studies (10, 11, 17, 20). 

This difference can be due to the presence of employed people in the community and gaining more awareness towards oral health than the housewives. In this study a significant correlation between CPITN index and frequency of tooth-brushing was seen. Zero code frequency in pregnant women who brushed 3 times a day was more than those who brushed one or two times a day and code 2 was more frequent in women who brushed only once a day. Golpasand, Torabi and Nouri also reported a significant correlation between CPITN and the frequency of tooth-brushing (10, 18, 20). 

In this study, although a significant relationship was not observed between CPITN and parity but in pregnant women who were in their first or second pregnancy, greater frequency of code 0 and code 1 of CPITN was observed and code 2 was more frequent in women who were in their third, fourth or fifth pregnancy. Torabi *et al* also observed the relationship between parity and CPITN (20). This may be due to the onset of the periodontal disease in the first pregnancy and being busier with children or even increasing maternal age at further pregnancies. In this study, CPITN increased as the month of pregnancy increased. The results of Yalcin *et al* study also reported that the plaque index, gingival index and pocket depth gradually increased in the period between the first trimester and the third trimester of pregnancy (11). Tezel also showed that the CPITN index, probing depth and gingival bleeding increases as gestational age increases (12). 

In conclusion, more than half of the pregnant women had healthy gums and the rest of them needed oral health instructions. Few patients required scaling and fortunately none required advanced surgical treatment. 

**Limitations**

Attraction of the cooperation of patients was one of our main limitations in this study.

## Note

***This article extracted from*** ***D.D.S. thesis. (Salem Mohammadi-Asl)***
